# Pressure-Enhanced Liquid Chromatography, a Proof of Concept: Tuning Selectivity with Pressure Changes and Gradients

**DOI:** 10.1021/acs.analchem.2c00464

**Published:** 2022-05-24

**Authors:** Szabolcs Fekete, Michael Fogwill, Matthew A. Lauber

**Affiliations:** † Waters Corporation, CMU-Rue Michel Servet 1, 1211 Geneva 4, Switzerland; ‡ Waters Corporation, 34 Maple Street, Milford, Massachusetts 01757, United States

## Abstract

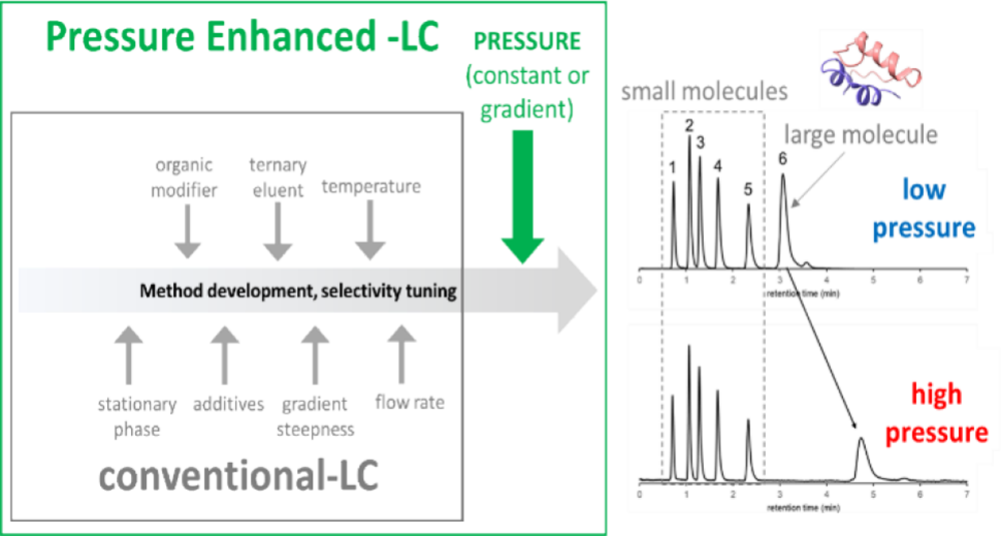

Many chromatographers have observed that the operating pressure can dramatically change the chromatographic retention of solutes. Small molecules show observables changes, yet even more sizable effects are encountered with large biomolecules. With this work, we have explored the use of pressure as a method development parameter to alter the reversed-phase selectivity of peptide and protein separations. An apparatus for the facile manipulation of column pressure was assembled through a two-pump system and postcolumn flow restriction. The primary pump provided an eluent flow through the column, while the secondary pump provided a pressure-modulating flow at a tee junction after the column but ahead of a flow restrictor. Using this setup, we were able to quickly program various constant pressure changes and even pressure gradients. It was reconfirmed that pressure changes impact the retention of large molecules to a much greater degree than small molecules, making it especially interesting to consider the use of pressure to selectively separate solutes of different sizes. The addition of pressure to bring the column operating pressure beyond 500 bar was enough to change the elution order of insulin (a peptide hormone) and cytochrome C (a small serum protein). Moreover, with the proposed setup, it was possible to combine eluent and pressure gradients in the same analytical run. This advanced technique was applied to improve the separation of insulin from one of its forced degradation impurities. We have referred to this method as pressure-enhanced liquid chromatography and believe that it can offer unseen selectivity, starting with peptide and protein reversed-phase separations.

## Introduction

In liquid chromatography (LC), it is well-known that the pressure drop on the chromatographic system (including the column) can significantly impact solute retention despite the fact that this effect is often considered to be negligible.^1−5^ Pressure-related effects are especially important in ultrahigh-pressure conditions and are more significant for large solutes.^6−11^ It is true that pressure-related effects often coexist with the development of friction-related heat gradients, which have a contrasting effect on retention compared to pressure.^10,11^ Therefore, if friction-related longitudinal thermal gradients are significant (e.g., a high flow rate is applied), then the pressure effects may remain invisible or minor, as the two effects compensate for each other.

For small-molecular-weight compounds, 10–50% increases in retention have been reported in isocratic separations when working with high-pressure conditions (e.g., in the 100–1000 bar pressure range).^3,4,6,12−14^ Retention changed to a higher extent when analyzing ionizable compounds or when working in ion-pairing (IP) conditions.^6,7,13^

On the other hand, for large molecules (e.g., peptides and proteins), a much greater influence on retention was reported when varying the operating pressure.^9,11,15,16^ With isocratic separations, retention increased by 150% for peptides (∼1.3 kDa), 800% for insulin (∼6 kDa), and up to >3000% for myoglobin (∼17 kDa) when increasing pressure from 100 up to 1100 bar.^9^ Composition-programmed gradient separations have also shown sensitivity to pressure as seen when analyzing intact monoclonal antibodies (mAbs) and their subunit fragments.^9^ In addition, a very interesting example was reported illustrating the impact of pressure on the change in the selectivity and resolution between insulin and related compounds (oxidized and reduced forms).^9^ Complete coelution occurred at a low pressure (*p* = 110 bar), while a higher than baseline resolution was achieved at a high pressure (*p* > 600 bar).

Pressure-related changes in retention are often explained by the change in the partial molar volume of the solute (Δ*V*_m_) associated with its transfer from one phase of the system to the other (e.g., the difference between molar volumes of the solute when adsorbed and desorbed).^17^ However, the change in the partial molar volume can be correlated with several phenomena taking place simultaneously during the adsorption–desorption process.^15^ For macromolecules, changes in Δ*V*_m_ may originate from various sources such as the variations in the energy of molecular interactions, solvation, aggregation, or changes in the energy of these interactions.^18^ Possible changes in the molecular conformation, caused by pressure, directly impact Δ*V*_m_ and can also modify the surface hydrophobicity of the solute molecule.^15^ The stoichiometric displacement model developed for protein separations predicts that the solute retention is a function of the number of solvent molecules that are displaced when the solute is adsorbed from the mobile phase onto the surface of the stationary phase.^19^ The folding or unfolding of large protein molecules upon adsorption is well-known in reversed-phase liquid chromatography (RPLC), and it is appreciated that those changes lead to exposure of the hydrophobic core and in turn an increase in solute retention. Conformational changes upon adsorption were reported in ion exchange (IEX) and in hydrophobic interaction chromatography (HIC) as well.^20−22^

All these observations suggest that operating pressure can be a useful variable to tune the selectivity of liquid chromatographic separations. However, pressure has not yet been comprehensively considered as a method parameter. Normally, in LC, pressure is constant throughout isocratic elution, but it varies slightly with composition-programmed gradient elution as a consequence of the viscosity differences of mixed solvent components. The purpose of this work was to study the possibility of using pressure as a method variable for altering the selectivity and resolution in LC. An instrumental setup is proposed here, which enables one to perform liquid chromatographic separations at arbitrary pressures. We were able to perform separations in constant pressure and in pressure-programmed gradient modes. Moreover, with the proposed setup, it is possible to combine composition-programmed and pressure-programmed gradients (i.e., a dual gradient mode), which improves the degrees of freedom for additional method development. Unique selectivity can be explored by purposefully changing the operating pressure. We believe that this new pressure-enhanced liquid chromatography (PE-LC) technique can open new possibilities and unseen selectivity, most especially for the reversed-phase separations of peptides and proteins.

## Experimental Section

### Chemicals and Samples

HPLC-grade water was obtained from Fisher Scientific (Dublin, Ireland). Acetonitrile, isopropanol (HPLC-grade), trifluoroacetic acid (TFA), uracil, methyl-paraben, ethyl-paraben, propyl-paraben, butyl-paraben, ketoprofen, human insulin, cytochrome C, and ribonuclease A were purchased from Sigma-Aldrich (Buchs, Switzerland). A mixture of test proteins (including ribonuclease A, cytochrome C, bovine serum albumin (BSA), myoglobin, enolase, and phosphorylase B) was manufactured by Waters and acquired in the form of a MassPREP Protein Standard Mix (Milford, MA, USA).

### Chromatographic System, Columns, and Software

All chromatographic separations were performed on a modified Waters ACQUITYUPLC H-Class Biobinary Plus system equipped with a binary solvent delivery pump, an autosampler with a flow-through needle (FTN), and a UV detector. The gradient delay volume was measured to be 115 μL, and the system’s extra column volume was measured to be 8 μL. In addition to this commercially available instrument setup, an extra binary delivery pump was also added to deliver a secondary solvent flow in order to control the pressure drop on the column (see more details in Apparatuses and Methodologies).

All experiments were performed on a 50 × 2.1 mm ACQUITY UPLC BEH 300 C4, 1.7 μm column (Waters, Milford, MA, USA). The column was equilibrated with a minimum of 20 column volumes of the mobile phase before injecting a set of samples.

The column backpressure was regulated by connecting a short capillary tube with a 25 μm I.D. and a length of 5 cm. The tube was connected between a T-junction unit (collecting liquid flows from the column and the extra pump) and a detector cell using a zero dead volume connector. The volume of the restrictor tube was negligible compared to the total extra column volume of the instrument. Therefore, the addition of a restrictor tubing of such a small volume prior to the detector was not expected to affect column efficiency and apparent retention. The capillary restrictor tube was purchased from SGE Analytical Science (Kiln Farm Milton Keynes, UK) (see more details in Apparatuses and Methodologies).

Data acquisition and instrument control were performed by Empower Pro 3 software (Waters, Milford, MA, USA). Calculations were performed in Microsoft Excel software and SigmaPlot software (Systat Software, Inc.).

### Sample and Mobile Phase Preparation

Test solution 1 (TS1) was a mixture of uracil, methyl-paraben, ethyl-paraben, propyl-paraben, butyl-paraben, and insulin diluted in water (100 μg/mL of each). TS1 was diluted from individual stock solutions of 10 mg/mL prepared in a mixture of acetonitrile, water, and TFA (10% + 90% + 0.1%, vol %). The sample was transferred to a polypropylene vial and injected directly.

Test solution 2 (TS2) was a mixture of insulin and cytochrome C (500 μg/mL of each) prepared in a mixture of acetonitrile, water, and TFA (10% + 90% + 0.1%, vol %). Forced degradation under thermal stress was performed by incubating the sample at 40 °C for two weeks. Then, samples were transferred to polypropylene vials and injected directly.

Test solution 3 (TS3) was a 500 μg/mL insulin solution prepared in a mixture of acetonitrile, water, and TFA (10% + 90% + 0.1%, vol %). The sample was transferred to a polypropylene vial and injected directly.

Test solution 4 (TS4) was a mixture of propyl-paraben, butyl-paraben, ketoprofen, and ribonuclease A (100 μg/mL of each) prepared in water. TS4 was diluted from individual stock solutions of 10 mg/mL prepared in a mixture of acetonitrile, water, and TFA (10% + 90% + 0.1%, vol %). The sample was transferred to a polypropylene vial and injected directly.

Test solution 5 (TS5) consisted of a MassPREP Protein Standard Mix reconstituted in 100 μL of 0.1% TFA in water, and it was injected directly.

For all measurements, mobile phase A was 0.1% TFA (v/v) in water, and mobile phase B was 0.1% TFA (v/v) in acetonitrile. Detection was carried out at 214 nm.

On the secondary pump, a postcolumn flow solvent (makeup solvent) comprised a highly viscous mixture of isopropanol and water (60% + 40%).

### Apparatuses and Methodologies

#### Instrumental Setup for Pressure-Enhanced Liquid Chromatography (PE-LC)

To perform accurate and independent pressure and column flow rate control, we considered an instrument setup that was proposed by Chester and Pinkston as a pressure-regulating fluid interface for supercritical fluid chromatography (SFC).^23^ In SFC, in most cases, flow control is applied on the upstream side of the column (precolumn), while pressure control is applied to the downstream (postcolumn) side (in the form of a backpressure regulator (BPR)). However, this setup is not ideal when low-pressure detectors (e.g., a mass spectrometer or light scattering) are used.^23^ To avoid pressure-related detector issues, the BPR was replaced with a tee junction, delivering a pressure-regulating makeup fluid from a separate, secondary pump. With such an instrument setup, the flow rate on the column and the mobile phase composition were controlled by the upstream pump (similarly to conventional SFC and LC), while the postcolumn pressure was controlled independently by the pressure-controlling pump, which was directed (through a tee junction) to a restrictor tube and then to the detector. Chester and Pinkston operated this secondary pump under pressure control (rather than flow control) to maintain a constant (200 bar) postcolumn pressure for their SFC applications. They used methanol as the pressure-regulating fluid. A similar setup was described by Takeuchi et al. where a low-pressure syringe pump was used to prevent boiling of the effluent in high-temperature liquid chromatographic (HTLC) applications.^24^

**Figure 1:**
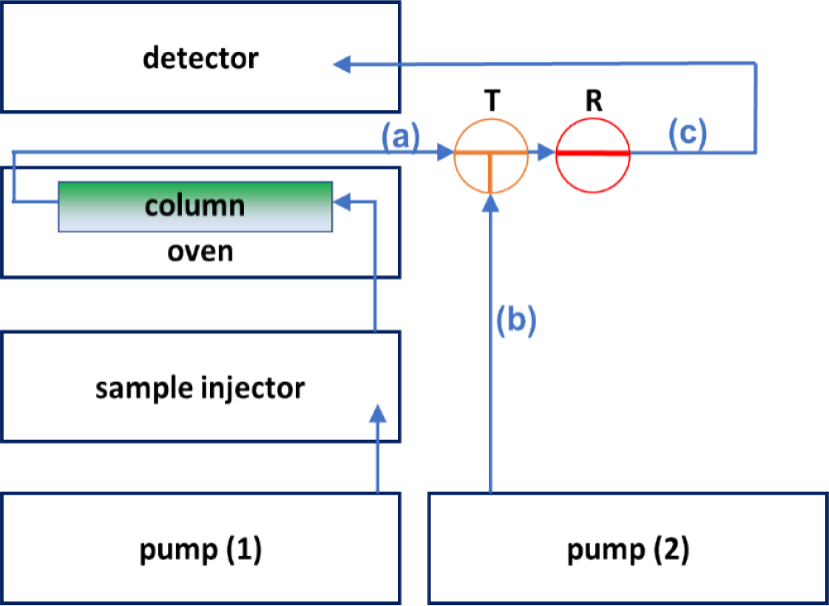
Schematic view of the pressure-enhanced liquid chromatographic (PE-LC) setup. Blue arrows indicate the direction of the solvent flow, T: tee junction, R: restrictor capillary. Capillaries (a) and (b) are made of stainless-steel of 100 μm I.D. (to resist very high-pressure conditions), while capillary (c) is a PEEK tubing of 100 μm I.D.

Our idea was to use a similar setup in LC. However, it was of interest to apply it for a different purpose, namely, to use pressure as a variable to change separation selectivity. We used an ultrahigh-pressure liquid chromatographic system (UHPLC) outfitted with an extra secondary pump (pump 2). Figure 1 shows a schematic view of our arrangement. The flow from the primary pump (pump 1) is directed through the column and then to a tee junction where the primary flow meets the pressure-regulating solvent flow. Then, the mixed fluid from the tee junction is directed to a short restrictor capillary tube (5 cm × 25 μm) and then to the optical (UV) detector. Placing the optical detector downstream of the pressure-controlling element offers the additional advantage of operating with a postcolumn pressure above the manufacturer-recommended detector flow cell pressure maximum of 100 bar, which would be the limit if the detector was placed upstream of the pressure-controlling element.

By changing the flow rate of the secondary pump, the column will experience different pressures (independently of the flow rate set on the primary pump). The flow rate and the mobile phase entering the column can be adjusted independently of the secondary flow. With such a setup, an arbitrary pressure can be set by changing the flow rate on the secondary pump. Moreover, if a flow rate gradient is programed on the secondary pump, then the column will experience a pressure gradient. Both positive and negative pressure gradients can be realized, and with such an arrangement, we are not limited to only linear pressure gradients. Concave, convex, or any multisegmented pressure gradients can be programmed. To the best of our knowledge, this is the first instrumental arrangement that allows an analyst to run LC or UHPLC separations at any pressure (up to 1000 bar; the pressure limit of the system) and any arbitrarily set pressure gradient profile.

#### Experiments Performed in the Constant Pressure Mode

When studying the impact of column pressure on retention and selectivity (in the isocratic elution mode), the postcolumn pressure was changed gradually by setting different flow rates on the secondary pump.

To separate the components of TS1, the following was performed. The primary pump was set to an *F*_1_ = 0.2 mL/min flow rate and 29% B mobile phase composition. The column temperature was equilibrated to *T* = 50 °C, and the flow rate of the secondary pump was set to a series of *F*_2_ = 0, 0.05, 0.10, 0.15, 0.20, 0.25, 0.30, and 0.35 mL/min (which covered *p* ∼ 250–730 bar pressure range).

Components of TS2 were separated with an *F*_1_ = 0.2 mL/min flow rate and a 31% B mobile phase composition. The column temperature was set to *T* = 50 °C, and *F*_2_ was set to 0, 0.10, 0.20, and 0.30 mL/min (which resulted in *p* ∼ 252, 393, 538, and 667 bar as the overall pressure drop).

#### Experiments Performed in the Pressure Gradient Mode

To see the precision of a pressure gradient (negative pressure gradient), we examined attempts to achieve linear, convex, and concave pressure gradients through several different flow rate programs on the secondary pump. The TS3 sample (insulin and its impurity) was used for this measurement.

An *F*_1_ = 0.2 mL/min flow rate and 29% B mobile phase composition (isocratic elution) were applied, and the column temperature was set to *T* = 50 °C. The *F*_2_ flow rate was changed from 0.30 to 0 mL/min across 10 min. Three different time programs were used: linear (1), convex (2), and concave (3) (in Empower instrument control software, they are gradient curve types 6, 2, and 10, respectively).

#### Combining Pressure and Solvent Gradients

Two other interesting approaches were also investigated, one to perform solvent gradients at various pressures (1) and another to combine solvent gradients with pressure gradients (2).

The TS4 sample was analyzed with these types of gradients. An *F*_1_ = 0.15 mL/min flow rate was set, and a 20–55% B in 25 min linear mobile phase composition gradient was applied. The column temperature was *T* = 70 °C. The *F*_2_ flow rate was set at different values (0, 0.10, 0.20, and 0.30 mL/min) to achieve solvent gradient measurements at various (constant) pressures (*p* = 190, 395, 480, 590, and 715 bar).

The TS2 sample was separated with a shallow solvent gradient as combined with either positive or negative pressure gradients. An *F*_1_ = 0.20 mL/min flow rate was set, and a 30–33% B in 6 min linear mobile phase composition gradient was run. The column temperature was equilibrated at *T* = 50 °C. The *F*_2_ flow rate was set at 0.25 mL/min to provide a reference separation (constant pressure mode), and then, two flow rate gradients were tested. The applied flow programs were *F*_2_ = 0–0.5 mL/min in 6 min (resulting in a linear positive pressure gradient, from 250 to 900 bar) and *F*_2_ = 0.5–0 mL/min in 6 min (resulting in a linear negative pressure gradient, from 900 to 250 bar).

#### Measuring Retention Model Parameters

It was of interest to study the change in retention model parameters as a function of operating pressure. Therefore, the TS5 sample was analyzed in the gradient elution mode at different (yet constant) pressures. Two different composition gradient slopes were studied. An *F*_1_ = 0.15 mL/min flow rate was set, and 20–55% B gradients were run in *t*_G1_ = 10 and *t*_G2_ = 20 min. The column temperature was controlled at a temperature (*T*) of 70 °C. The *F*_2_ flow rate was set at different values (0, 0.10, 0.20, 0.25, and 0.30 mL/min). The column hold-up time was measured by injecting uracil as a *t*_0_ marker.

A commonly used semiempirical retention model was assumed (linear solvent strength model, LSS) to estimate the change in retention as a function of eluent composition. The *S* model parameter expresses the sensitivity of solute retention changes *vs* mobile phase composition. , where *k* is the retention factor of the solute, *k*_0_ is the retention factor measured in the weakest mobile phase (e.g., mobile phase A), and φ is the volume fraction of the stronger eluent in the actual mobile phase. The *S* parameter was determined at various pressures. The *S* model parameters at each pressure were derived from two composition gradient experiments by using a linear fitting procedure.^25−27^ Then, *S* parameters were plotted as a function of pressure for all seven proteins, and polynomial curves were fit to illustrate the trends.

## Results and Discussion

### Separations at Various Constant Pressures

Pressure effects have already been extensively studied in the constant pressure mode using restrictor capillaries of different lengths to tune the outlet pressure after the column. However, our setup offers the benefit that pressure can be set at will without reconfiguring the system and thus can be used as a continuous (nondiscrete) variable for method development. A short summary is provided here about constant pressure experiments.

**Figure 2:**
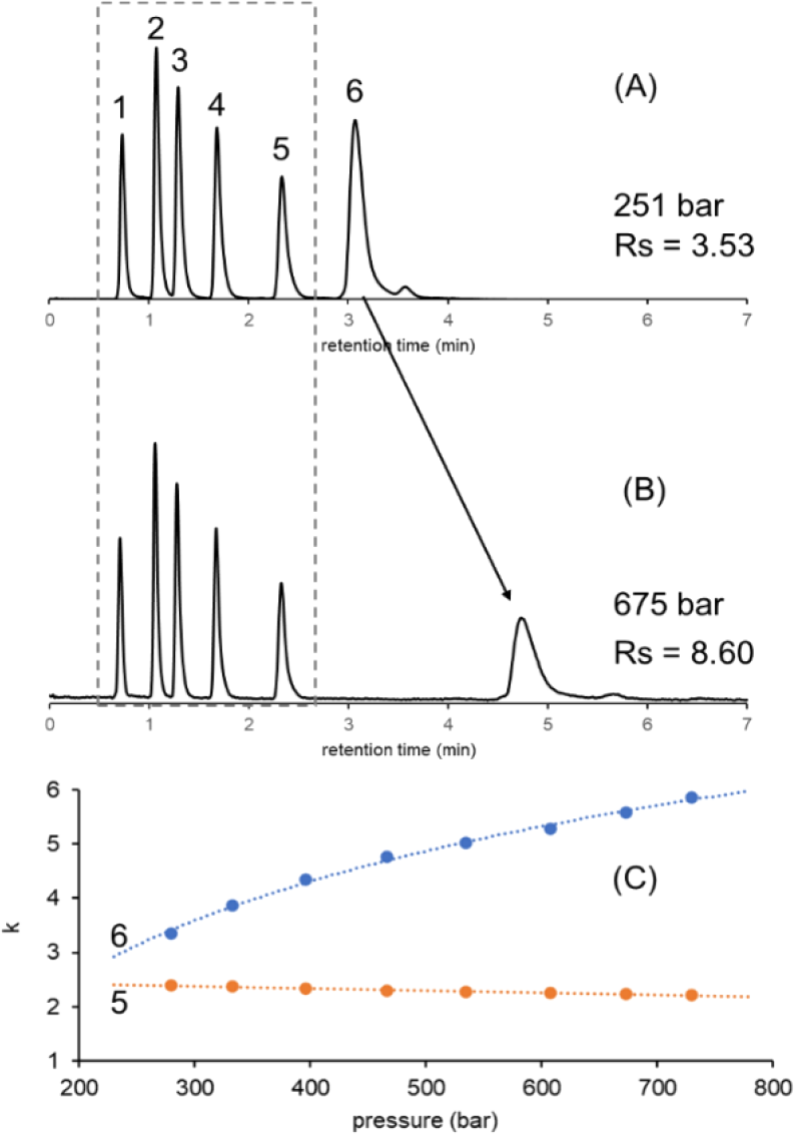
Separation of small molecules (parabens) and a moderate-size molecule (insulin) in the isocratic elution mode at a low pressure (A) and at a high (B) pressure and the change in retention (*k*) as a function of the column inlet pressure (C). Peaks: uracil (1), methyl-paraben (2), ethyl-paraben (3), propyl-paraben (4), butyl-paraben (5), and insulin (6).

Figure 2A,B shows two representative chromatograms obtained for the mixture of parabens and insulin in an isocratic separation. The resolution between butyl-paraben and insulin was *R*_s_ = 3.53 when operating the column at an inlet pressure (*p*) of 251 bar (only intrinsic column pressure drop) (Figure 2A). Then, when increasing the operating pressure by gradually changing the flow rate delivered by the secondary pump, the retention of insulin was seen to continuously increase, while the retention of the parabens remained nearly the same. Figure 2C plots the retention factor (*k*) as a function of the column inlet pressure for butyl-paraben and insulin. As expected, the retention of insulin showed a dramatic effect versus the retention of butyl-paraben.

In light of this, it can be recognized that a new kind of selectivity can be attained by changing column pressure. Moreover, the selectivity changes accessible by pressure effects are not expected to be the same as those obtained through traditional method variables, such as eluent strength and mobile phase temperature. The use of pressure is especially interesting when solutes of different sizes (masses) are needed to be separated.

**Figure 3:**
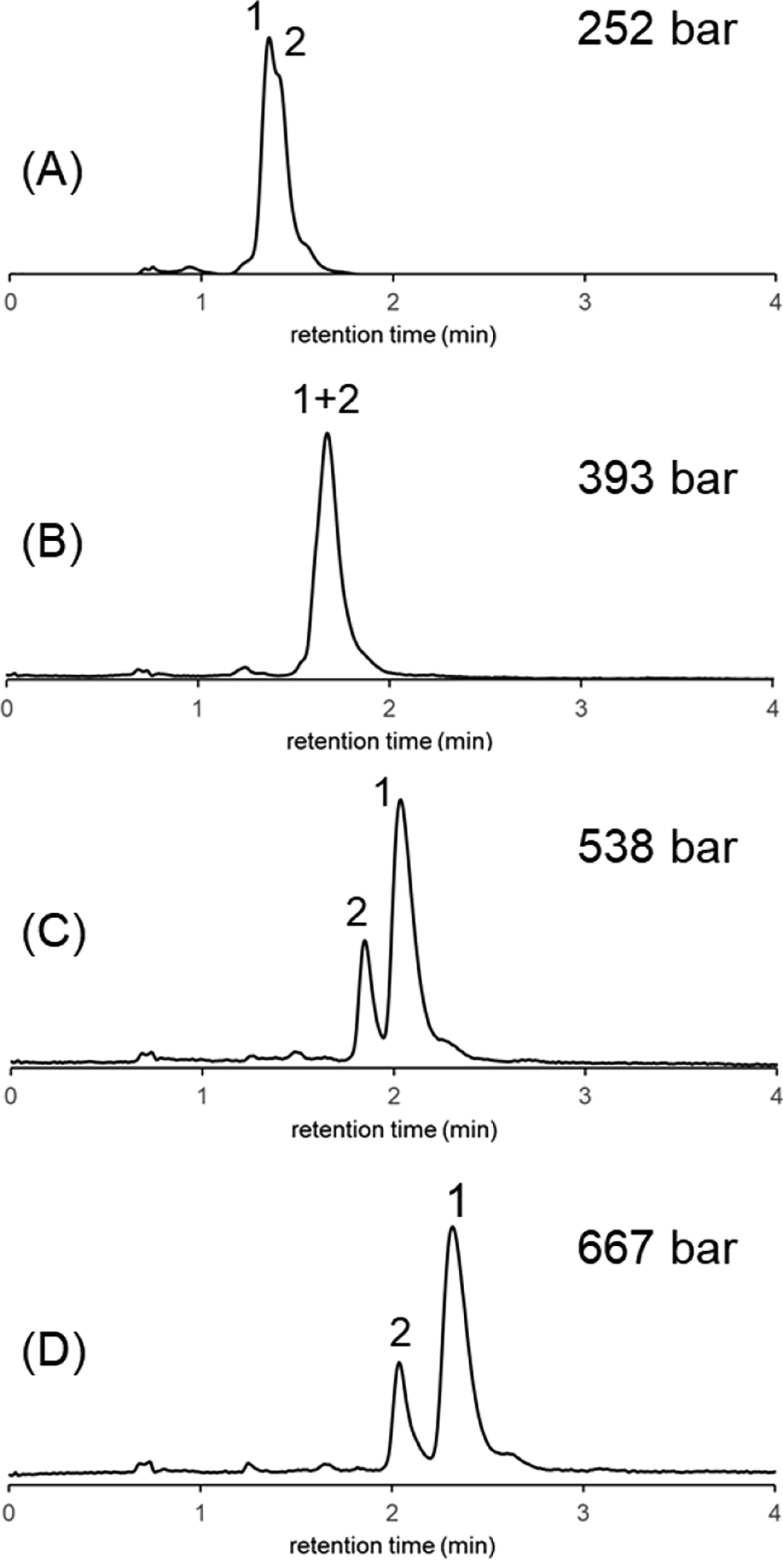
Separation of cytochrome C and insulin in the isocratic elution mode at various pressures. Experiments were performed at *p* = 252 (A), 393 (B), 538 (C), and 667 bar (D) (whose values correspond to average column pressures that solutes experience at the middle of the column, which are *p* = 126, 267, 412, and 541 bar, respectively). Peaks: cytochrome C (1) and insulin (2).

Another example is shown in Figure 3. In this case, the evolution of selectivity between two large solutes (insulin and cytochrome C) was studied. The molecular weight of cytochrome C (*M* ∼ 12.4 kDa) is about twice that of insulin (*M* ∼ 5.7 kDa). Accordingly, it was predicted that adding pressure to an isocratic separation would most significantly impact cytochrome C. Indeed, by increasing operating pressure, the retention of cytochrome C increased to a larger extent compared to the retention of insulin. The addition of pressure to bring the column inlet pressure beyond 500 bar was enough to change the elution order of the two components. This example shows that pressure alone can give rise to elution order changes.

### Performing Linear and Nonlinear Pressure Gradients

Since, in a certain sense, pressure has a similar effect on solute retention to eluent strength, we were motivated to perform pressure gradient separations (similarly to a common mobile phase gradient separation). First, we considered a negative pressure gradient since it is the most similar mode to a solvent gradient. When decreasing pressure, solute retention is expected to decrease. Therefore, we tried to perform negative linear, convex, and concave pressure gradients by setting linear, convex, and concave flow rate programs in the secondary pump.

**Figure 4:**
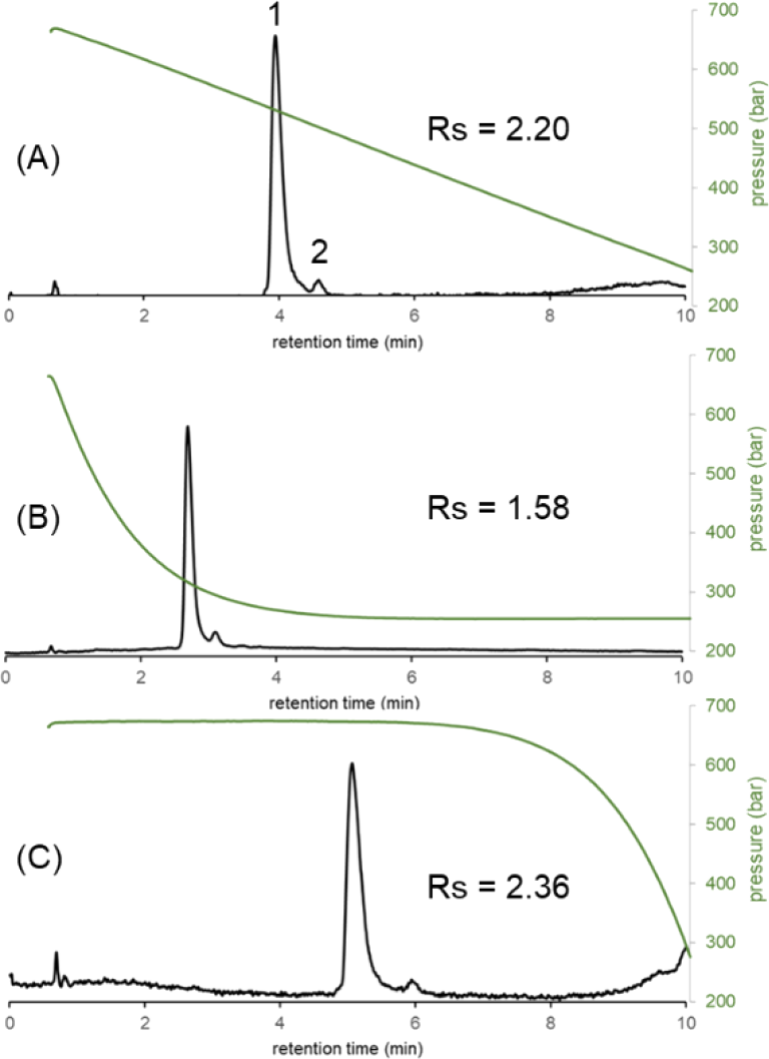
Chromatograms of insulin and an insulin impurity obtained in linear (A), convex (B), and concave (C) pressure gradient modes, when an isocratic elution mode was applied. The green curves show the experimentally measured pressure gradients. Peaks: insulin (1) and an insulin impurity (2). Figure S1 (Supporting Information) shows the evolution of pressure as a function of the time and the column length in the case of a convex pressure gradient.

Figure 4 shows the chromatograms obtained for insulin and an insulin impurity in three different pressure gradient modes. The overlayed green curves show the experimentally measured pressure gradients. It seems that with our active flow control setup, very precise linear and nonlinear pressure gradients can be performed.

It would also seem to make sense to perform both linear and nonlinear pressure gradients, specifically because the peak resolution was significantly different in the three pressure gradient modes. The resolution varied between *R*_s_ = 1.58 and 2.36 depending exclusively on the shape of the pressure gradient program (the initial and final pressures as well as the gradient time were fixed). It is also worth mentioning that the peak width depends on the rate of the pressure gradient (similarly to solvent gradients). A kind of pressure gradient band compression is observed when working in the negative pressure gradient mode. The steeper is the pressure gradient, the thinner is the peak. Of course, this effect was only observed with large-MW solutes. On the contrary, when running positive pressure gradients, band expansion (peak broadening) was observed.

### Changing Pressure in the Gradient Elution Mode

Most large-molecule LC separations are performed with gradient elution. Thus, we were interested in studying the possibilities of PE-LC in the gradient elution mode too.

**Figure 5:**
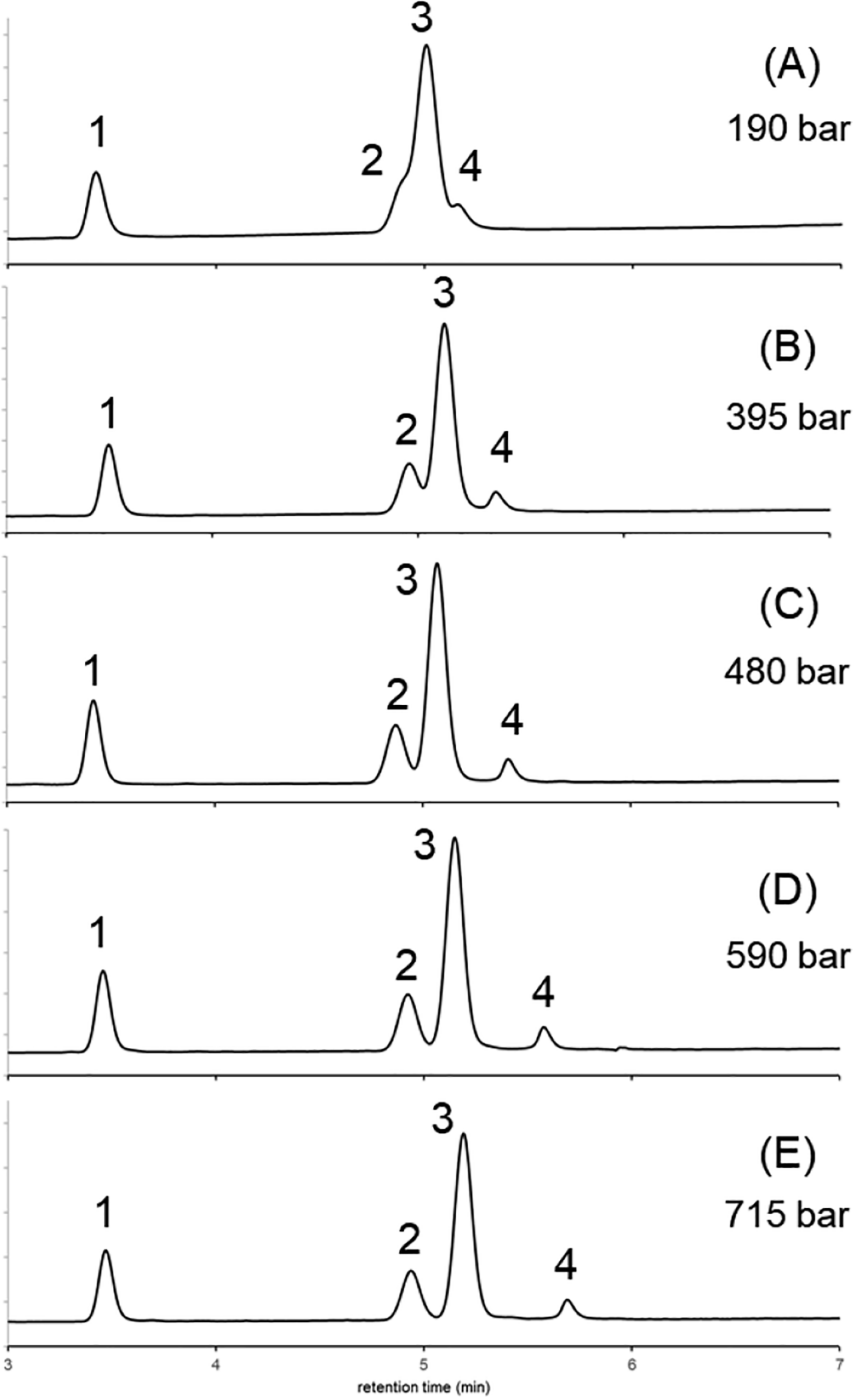
Gradient elution separations performed at constant but different pressures. Experiments were performed at *p* = 190 (A), 395 (B), 480 (C), 590 (D), and 715 bar (E) (whose values correspond to average column pressures that solutes experience at the middle of the column, which are *p* = 95, 300, 385, 495, and 620 bar, respectively). Peaks: propyl-paraben (1), butyl-paraben (2), ketoprofen (3), and ribonuclease (4).

First, we worked with constant postcolumn pressures and performed solvent gradient separations at different column pressures. Figure 5 shows the obtained chromatograms. The sample contained propyl-paraben, butyl-paraben, ketoprofen, and ribonuclease (a mixture of small and large solutes). As observed, the largest compound (ribonuclease A, *M* = 13.7 kDa) showed the most important retention shift when increasing the operating pressure. The second largest solute (ketoprofen, *M* = 0.254 kDa) showed a moderate shift, while the smallest analyte (butyl-paraben, *M* = 0.194) remained nearly unchanged. Therefore, by increasing the column pressure of this solvent gradient separation, the elution window of this peak triplet was significantly widened, and the resolution was drastically improved.

**Figure 6:**
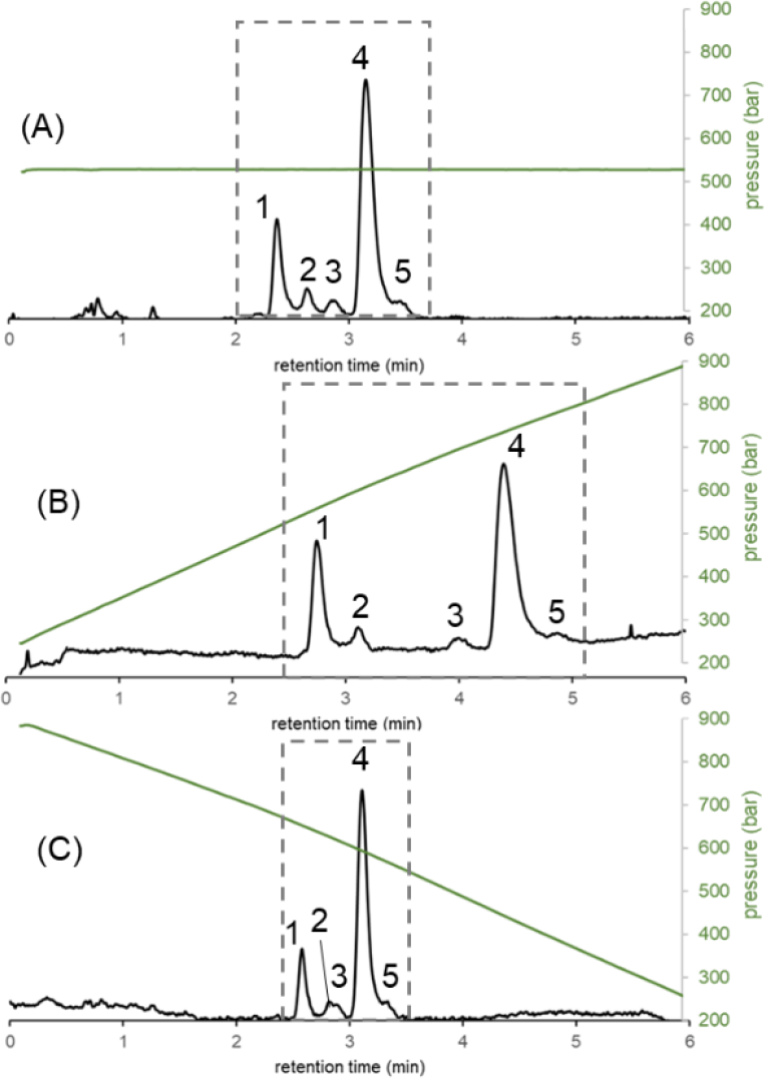
Gradient elution separations performed in the constant pressure mode (A) and in positive linear (B) and negative linear pressure gradient (C) modes. The green curves show the experimentally measured pressure gradients. Peaks: insulin (1), an insulin impurity (2), a cytochrome C impurity (3), cytochrome C (4), and a second cytochrome C impurity (5). Sample: a forced degradation sample.

In the next example, we performed solvent and pressure gradients simultaneously. Figure 6 shows the separation of insulin-related and cytochrome C-related peaks (large solutes) as eluted with different pressure gradient profiles. As can be seen, a positive pressure gradient significantly stretched the elution window, while a negative pressure gradient resulted in elution window compression. Therefore, selectivity is expected to increase when running positive pressure gradients, albeit with peaks slightly broadened by band decompression.

### Impact of Pressure on Retention Model Parameters

To better understand PE-LC, some fundamentals have been examined. Since large molecules tend to exhibit on–off elution behavior,^25−27^ the retention of large molecules is therefore very sensitive to the mobile phase composition (eluent strength). Accordingly, a very minor change in mobile phase composition can result in complete release of the solute from the column. This on–off behavior is the main reason why the gradient elution mode is preferred for large-molecule separations. In the LSS model, the *S* parameter measures this retention sensitivity. For small molecules, normally, *S* < 10, while the on–off like behavior of large molecules starts at *S* ≥ 20–25. This *S* parameter was studied as a function of pressure.

**Figure 7:**
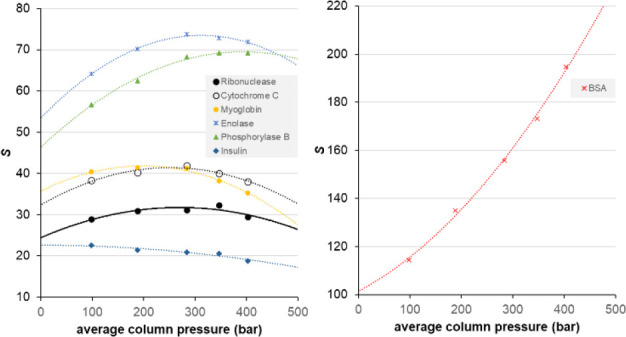
Plots of the *S* parameter *vs* average column pressure (that solutes experience at the middle of the column) for a series of compounds (left) and BSA (right).

Figure 7 shows the change in *S* values in a pressure window of *p* = 1–500 bar for various proteins covering a broad range of molecular weights (5.7 ≤ *M* ≤ 97 kDa). (Please note that here, the average column pressure is plotted, which is more practical for model calculations, and not the observed inlet pressure. The pressure drop across the column was 196 bar.) Based on these trends, the impact of pressure is observed to be protein-dependent. This is logical since different kinds of proteins (e.g., globular or linear) will undoubtedly show different pressure-induced conformational changes. In some cases, proteins are described to be structurally “softer”.^22^ Softer proteins possess more compressible structural motifs and are prone to unfolding. Behavior like this probably has a major impact on retention and pressure sensitivity.

Most proteins showed a local maximum on their *S vs* pressure plots, suggesting some structural transition in the studied pressure range. On the other hand, insulin (a peptide hormone and the polypeptide in this sample) showed a continuous decrease in its *S* parameter with pressure. On the contrary, the *S* parameter of BSA showed a continuous increase when increasing pressure. Each of these results suggests that the pressure dependence of retention is protein-dependent and needs to be measured individually. This step should be part of the optimization of future combination solvent and pressure gradient methods.

### Robustness, Reliability of Pressure, and Mobile Phase Composition

Please note that a high-pressure mixing binary pump was used. Binary pumps have compressibility compensation; therefore, there should be consistent accuracy in volumetric delivery. Due to the different rates of solvent compressibility (e.g., acetonitrile is more compressible than water), the mobile phase composition will be slightly impacted by pressure. This pressure-induced compositional difference should result in an increase in % B (stronger solvent) at increased pump pressures due to the higher compressibility of acetonitrile. Therefore, large solutes would have decreased retention time at high pressures compared to low pressures, if the pressure-induced compositional difference was the dominant variable. However, in our experiments on large solutes (on–off behavior), we have exclusively observed increased retention at higher pressures. This suggests that pressure effects overcome the very minor effect of nonideal solvent mixing (if there is any).

## Conclusions

A new approach (pressure-enhanced liquid chromatography (PE-LC)) has been proposed, which utilizes pressure as a method variable to change the selectivity of liquid chromatographic separations.

A two-pump LC system was used, in which the primary pump controlled the column flow rate and the mobile phase composition, while the secondary pump controlled the pressure drop on the system. With this arrangement, it was possible to perform measurements at any (arbitrary) pressure (up to *p* = 1000 bar, the system maximum pressure). In addition, precise pressure gradients (both linear and nonlinear) could be realized.

It seems that large-molecule separations or mixtures of small and large solutes could benefit the most from PE-LC. Pressure alone can change selectivity and thus the peak resolution. Increases in operating pressure can also induce retention order changes.

When performing pressure gradient separations, positive or negative slope gradients can be applied, though the shape of the pressure gradient program should be carefully considered in order to optimize the resolution.

The PE-LC approach also enables an analyst to simultaneously combine mobile phase and pressure gradients. This mode matches best with large-molecule separations. In this combined mode, a positive pressure gradient yielded a stretched elution window. Meanwhile, a negative pressure gradient produced a compressed one. Therefore, selectivity was observed to increase when running positive pressure gradients; however, a negative pressure gradient can be beneficial with respect to band compression and sharpening peaks.

Based on our preliminary work, the pressure sensitivity of retention increases with the size of the solute; however, this phenomenon is sample-dependent. Different proteins show different pressure effects such that it may be necessary to empirically optimize pressure-enhanced LC methods.

In the end, we believe that this novel PE-LC approach opens new possibilities in liquid chromatography, especially for samples containing solutes of moderate to large sizes. Finally, it is worth mentioning that with the PE-LC approach, unique selectivity can be obtained by intentionally changing the operating pressure. Such changes in selectivity cannot be achieved by changing other common method variables, like mobile phase composition and temperature. Introducing pressure as a method variable will increase the degrees of freedom for method development strategies.

## Supporting Information Available

Evolution of pressure as a function of the time and the column length in the case of a convex pressure gradient (PDF)

## Notes

Waters, ACQUITY UPLC, BEH, and Empower are trademarks of Waters Technologies Corporation. Excel is a trademark of Microsoft Corporation, and SigmaPlot is a trademark of Systat Software, Inc.
