# Supplementary material for: Pressure-Enhanced Liquid Chromatography, a Proof of Concept: Tuning Selectivity with Pressure Changes and Gradients
Source: Anal Chem. 2022 May 24;94(22):7877–84. doi: 10.1021/acs.analchem.2c00464 (PMC9178557; doi:10.1021/acs.analchem.2c00464)
Supplement: Supplementary file 1 — ac2c00464_si_001.pdf [file ac2c00464_si_001.pdf]

Supporting Information

Pressure-Enhanced Liquid Chromatography, a Proof of Concept: Tuning Selectivity with Pressure Changes and Gradients

Szabolcs Fekete[1*], Michael Fogwill,[2] Matthew A. Lauber[2]

[1] Waters Corporation, located in CMU-Rue Michel Servet 1, 1211 Geneva 4, Switzerland

[2] Waters Corporation, 34 Maple Street, Milford, MA 01757, United States

Table of Content

Figure S1. Evolution of pressure along a column in a PE-LC system S1

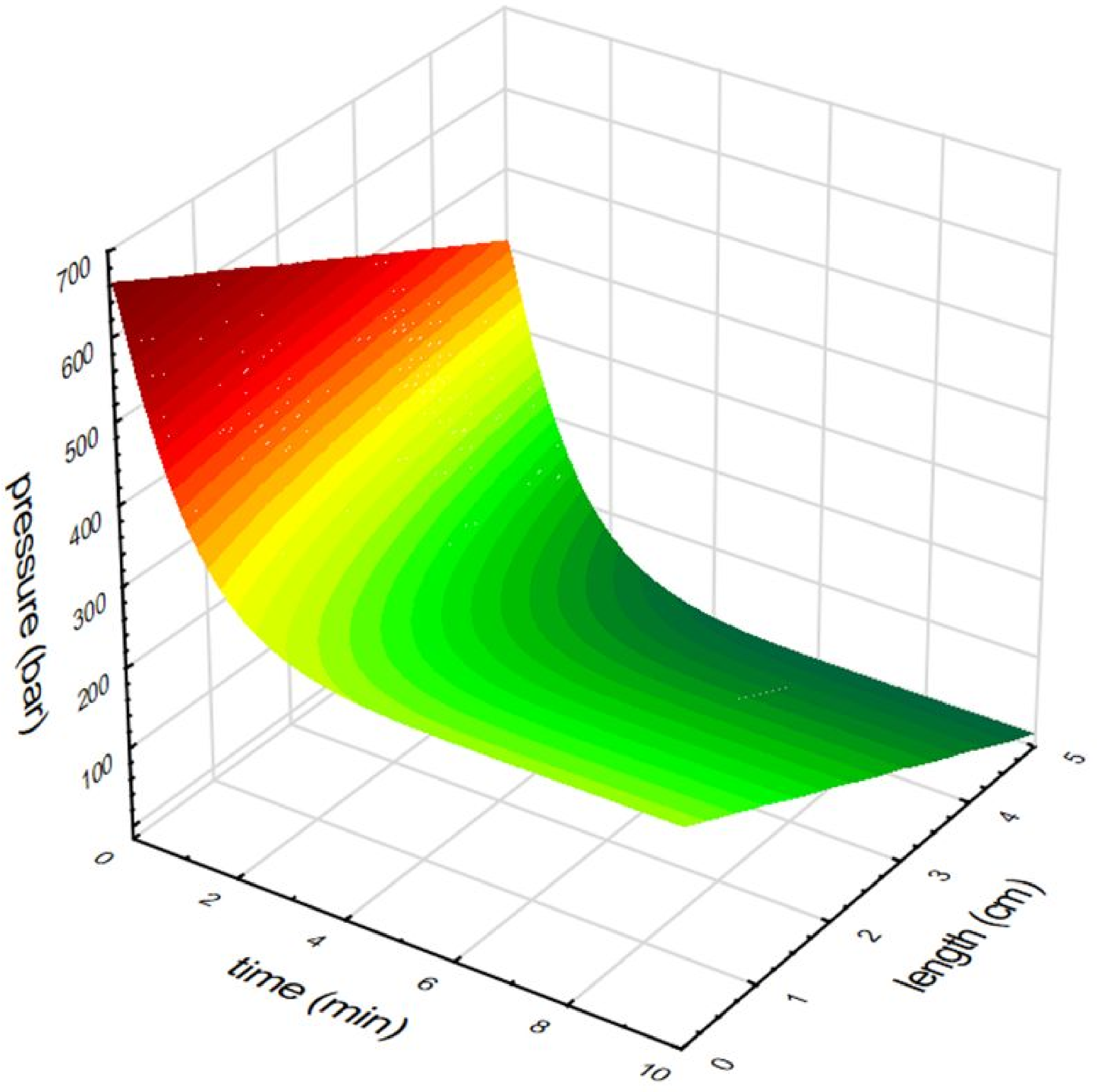

Figure S1. The evolution of pressure as function of time (t_G = 10 min) and column length (L = 5 cm) in case of a convex pressure gradient. The values at L = 2.5 cm correspond to the "average column pressure".
